# Origins of Cell-to-Cell Bioprocessing Diversity and Implications of the Extracellular Environment Revealed at the Single-Cell Level

**DOI:** 10.1038/srep17689

**Published:** 2015-12-14

**Authors:** A. E. Vasdekis, A. M. Silverman, G. Stephanopoulos

**Affiliations:** 1 Department of Physics, University of Idaho, Moscow, ID, 83844, USA; 2 Environmental and Molecular Sciences Laboratory, Pacific Northwest National Laboratory, Richland, WA, 99354, USA; 3 Department of Chemical Engineering, Massachusetts Institute of Technology, Cambridge, MA 02139, USA

## Abstract

Bioprocess limitations imposed by microbial cell-to-cell phenotypic diversity remain poorly understood. To address this, we investigated the origins of such culture diversity during lipid production and assessed the impact of the fermentation microenvironment. We measured the single-cell lipid production dynamics in a time-invariant microfluidic environment and discovered that production is not monotonic, but rather sporadic with time. To characterize this, we introduce bioprocessing noise and identify its epigenetic origins. We linked such intracellular production fluctuations with cell-to-cell productivity diversity in culture. This unmasked the phenotypic diversity amplification by the culture microenvironment, a critical parameter in strain engineering as well as metabolic disease treatment.

The emerging paradigm of constructing target phenotypes for the production of chemical products and biofuels is attracting considerable interest and has met with significant success in recent years^1^,^2^,^3^,^4^. Despite substantial progress, however, engineered strains in culture do not typically reach the theoretical maximum, questioning thus the strain’s phenotypic robustness. Additionally, single cell analysis reveals a considerable productivity variance within a clonal population. Whereas few individual cells greatly outperform the median productivity, others lag in productivity and are even less efficient than non-engineered strains. This in essence limits our ability to register the true phenotype of a construct and, as such, it limits its bioprocessing reliability.

Here we investigate the aforementioned limitations in bioprocesses at the single cell level with a specific focus on the *de-novo* lipid biogenesis of *Yarrowia lipolytica*, a promising candidate in the production of oleochemicals^5^,^6^,^7^. First, we determined the phenotypic diversity in culture during batch growth of an over-producing and an under-producing strain. Subsequently, the lipid production dynamics of both strains were analyzed at the single cell level using microfluidics, primarily for two reasons. The first was to perform longitudinal investigations by tracking the lipid content at the single cell level over time. The second was to deterministically control the extracellular hydrodynamics, thus generating a quasi-time-invariance through ultra-fast nutrient supply and byproduct removal. Under such conditions, the inherent intracellular fluctuations of lipid abundance within an ideal extracellular environment were unmasked. We identified the epigenetic origins of such fluctuations and then compared them to the lipid content diversity between individuals in culture, similar to recent gene expression noise investigations^8^,^9^,^10^,^11^,^12^. This revealed that extracellular microheterogeneities during fermentation amplify intracellular fluctuations, thereby placing an upper limit in the culture’s productivity and bioprocessing reliability.

## Results

**Figure 1:**
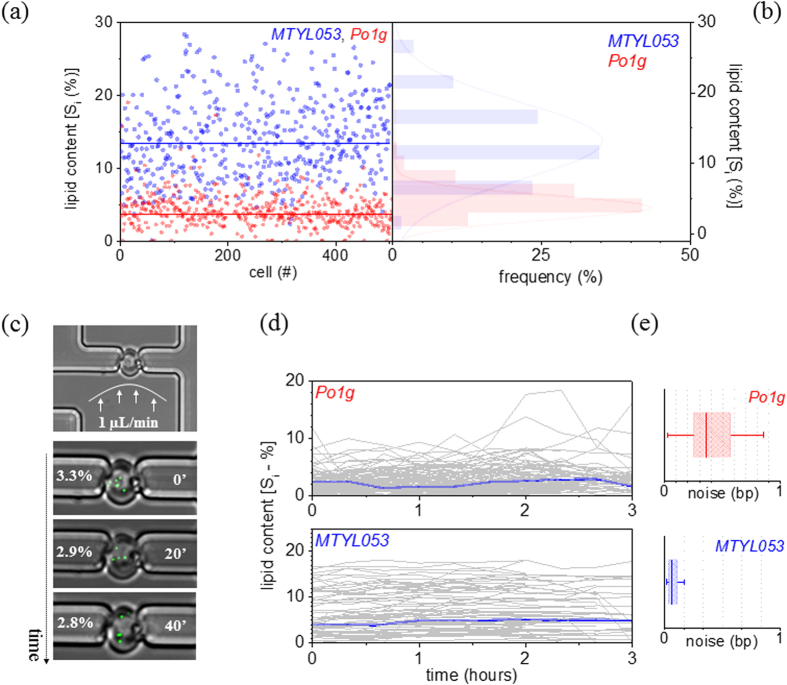
(**a**) A scatter plot illustrating the neutral lipid content normalized over the cell area for individual *Po1g* (red) and *MTYL053* (blue) cells; the median lipid content for each strain is represented with a solid line. (**b**) The frequency distribution of the lipid content per cell for the two strains, (**c**) Microfluidic immobilization of single *Po1g* cells under continuous laminar flow at a 1 μL/min rate (*top*). In the lower snapshot series at 20′ time-steps, composite images of the cell (bright-field) and its neutral lipid load (green) illustrate the fluctuation of the cell’s lipid content (S_i_). (**d**) Longitudinal traces of the lipid content per cell for *Po1g* (*upper*) and *MTYL053* (*lower*); the lineage of an individual cell is highlighted in blue. (**e**) Boxcharts (range: 25–75%) illustrating the single-cell bioprocessing noise for *Po1g* (*upper*) and *MTYL053* (*lower*); the whisker lines denote the outliers.

*Phenotypic Diversity in Culture:* The *Po1g* and *MTYL053* strains of *Yarrowia lipolytica* were investigated, as previously developed and extensively characterized^5^. *Po1g* and *MTYL053* are identical except for leucine auxotrophy in *Po1g* and an intron-containing translation elongation factor-1α (TEF) promoter for over overexpressing diacylglycerol acyltransferase (DGA1) in *MTYL053*. As a result, the expression levels of DGA1 in *MTYL053* are 17-fold enhanced compared to the intronless TEF promoter, as previously measured in detail^5^ (also qualitatively shown in Fig. S1). DGA1 is the final step of the triglyceride synthesis pathway thereby enabling *MTYL053* with enhanced lipid production^5^. To evaluate the phenotypic diversity in culture, image cytometry^13^ was employed (Supplementary Information) to define the size and number/size of lipid droplets (LDs) in individual cells. As shown in Fig. 1a, the median lipid content (S_i_—normalized over the cell size) during early stationary phase (Fig. S2) was 4.1% for *Po1g* and – as expected – considerably higher for *MTYL053* (14.2%).

Despite their different productivities, both strains suffered from a considerable lipid content diversity. This is best depicted by the distributions in (Fig. 1b), denoting the co-existence of individuals with lipid content lower or higher than the median for both strains. The phenotypic diversity was determined through the robust coefficient of variation (rCV—Supplementary Information), defined as the ratio of the robust standard deviation over the median. The rCV was employed to define the deviation of individual cells from the population’s median, instead of conventional estimates of central tendencies (mean) and dispersion (standard deviation). The latter assume the data form a random sample from a normal distribution. This is not the case in our experiments due to the presence of outliers as typically observed in single-cell analyses, such as the recent lipid content heterogeneity investigations using flow cytometry^14^. Under the aforementioned conditions, the rCV is similar to phenotypic robustness and thus linked to strain reliability (R) through the relationship: *R* = *1 – rCV*^15^,^16^. The reliability factors for *Po1g* was 0.31 and moderately improved for *MTYL053* (0.5). Indicative of the similar reliability factors is the considerable phenotypic overlap of *MTYL053* with the non-engineered *Po1g* (Fig. 1b). This questions our ability to register the true phenotype of the engineered strain, as well as the reliability of the pathway engineering strategy^17^.

*Single-Cell Lipid Production Dynamics:* To investigate this phenotypic overlap, we performed a time-dependent analysis of lipid production at the single cell level. Both single *Po1g* and *MTYL053* cells were immobilized using microfluidics (Fig. 1c) and their lipid content was measured every 20 min using confocal microscopy and vesicle photonics^18^ (Supplementary Information). The protein content was not simultaneously monitored in this instance, in order to avoid perturbations to cell physiology (e.g. cell lysis for single-cell qRT-PCR), or temporal resolution limitations in wavelength-multiplexed confocal imaging due to the high variability in maturation times of fluorescent proteins^19^. During imaging, laminar microflows (1 μL/min rates) were applied, enabling rapid replenishment of the extracellular environment at frequencies greater than 10 cell volumes/msec. This generated a quasi-time invariant extracellular microenvironment, a critical aspect in our study given the rapid metabolome dynamics with perturbation response times as low as 1 sec or less^20^. The employed flow rates also applied mechanical forces on the cells; however, these are in the 10 pN range, therefore exerting insignificant mechanical stress given the enhanced surface modulus of yeast^21^. Prior to microfluidic immobilization, cells were harvested at early stationary phase, thus enabling direct comparison with the aforementioned phenotypic diversity analysis, as well as prolonged viability, enhanced lipid content and low budding probability (Supplementary Information and Fig. S3, S4).

Under such conditions, lipid accumulation in either strain was not monotonic, but rather fluctuated with time through continuous cycles of replication and degradation (Fig. 1d). This unmasks an inherent form of noise in biosynthesis, termed *bioprocessing noise* (*bp*). Similar forms of noise have been previously defined in gene expression^8^,^22^,^23^,^24^,^25^,^26^,^27^,^28^ and recently in cell growth^29^ and metabolic pathway use^30^. We attribute the observed noise during biosynthesis to the sporadic nature of both gene expression and metabolic reactions. In essence both types of reactions exhibit an efficiency that depends on the abundance, activity and locations of all involved reactants, namely regulatory elements during gene expression, as well as metabolites, enzymes and co-factors during metabolic synthesis or degradation. Such copy-number and state fluctuations give rise in turn to a probability distribution of the reaction product itself. Similar to the nomenclature of gene expression noise^8^,^22^,^23^,^24^,^25^,^26^,^27^,^28^, the ‘extrinsic’ and ‘intrinsic’ contributions to bioprocessing noise emanate from variability in enzyme production and variability at the metabolic reaction level respectively. The single-cell bioprocessing noise was quantified through the robust coefficient of variation of the lipid content longitudinal trace (Fig. 1d). The median noise for *Po1g* was bp_*Po1g*_ = 0.35, while the transformed strain *MTYL053* exhibited substantially less noise by approximately 6-fold (bp_*MTYL053*_ = 0.06) (Fig. 1e).

## Discussion

**Figure 2:**
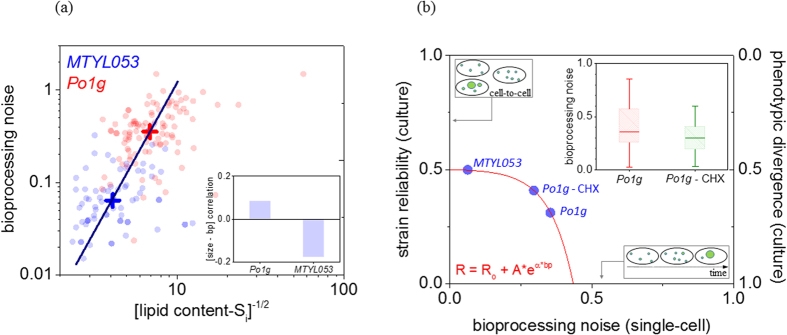
(**a**) Bioprocessing noise as a function of the inverse square root of lipid content; each data point denotes the median per cell for *Po1g* (red) and *MTYL053* (blue) with the larger cross-points indicating the median per strain interconnected through a linear fit. Inset histogram plots the correlation coefficients between bioprocessing noise with cell size for both *Po1g* and *MTYL053*. (**b**) The non-linear dependence of population level reliability (R) and phenotypic diversity on bioprocessing noise (bp) for *Po1g*, *Po1g* + CHX and *MTYL053*; the red line represents the exponential decay fit of the form: R = R_o_ + A∙e^α∙bp^, where R_o_ = 0.5, A = −0.003 and α = 12. Inset compares the bioprocessing noise box-charts for *Po1g* and *Po1g* + CHX.

It becomes apparent that enzyme over-expression in *MTYL053* not only induces enhanced lipid production but also reduces bioprocessing noise. This is better visualized in Fig. 2a that plots the median lipid content and bioprocessing noise between the two strains (cross points). An analogous dependence also persists within individual cells in each strain (Fig. 2a – scatter plot), with bioprocessing noise scaling inversely with the square root of lipid content in both strains. Interestingly, bioprocessing noise was independent of cell size for both strains, eliminating thus growth rate or stage variations as possible sources of the observed noise differences between individuals (Fig. 2a-inset).

It is relevant to compare the aforementioned inverse scaling with a similar one frequently encountered in gene expression noise^8^,^22^. Therein, gene expression noise also scales inversely with the square root of protein abundance, especially for proteins of low and medium abundances. This is associated with the spontaneous rate fluctuations in gene expression, emanating from micro- heterogeneities and crowding of the cytosol and cell-cycle dependent protein copy numbers^8^. Our interpretation of the above in bioprocesses is that in addition to gene expression, metabolic reactions are also subject to spontaneous rate fluctuations. Consequently, a continuous probability distribution within individual cells for both enzyme and metabolite copy numbers is enforced, contributing thus to the overall bioprocessing noise.

To compare the bioprocessing noise contributions of gene expression and metabolic reaction fluctuations, we added cycloheximide (CHX) to the YPD media (33 μg/ml) and repeated the dynamic analysis on the noisiest strain (*Po1g*). CHX terminates eukaryotic protein synthesis (and inhibits growth Fig. S2), thus diminishing noise contributions during transcription or translation^31^. Indeed, the addition of CHX reduced bioprocessing noise by approximately 15% in *Po1g* (Fig. 2b – inset). This evidences that gene expression fluctuations do contribute to bioprocessing noise. Bioprocessing noise however is not completely diminished under CHX admission, demonstrating that contributions from metabolic reaction fluctuations are also present and likely more dominant. It is worth noting that the randomness of gene expression and metabolic reactions are known to follow an epigenetic/non-Mendelian heredity pattern^8^. This indicates that bioprocessing noise is an intrinsic characteristic of a culture, bounded largely by enzyme expression levels as evidenced by the *Po1g*—*MTYL053* comparison.

The question however persists: if bioprocessing noise is lower in *MTYL053* than *Po1g* in microfluidics, then why do the two strains exhibit comparable reliability in culture during batch growth? To answer this, we plotted the reliability factor (R) of all three cases (*Po1g*, *Po1g-CHX* and *MTYL053*) against bioprocessing noise (bp) in the same graph (Fig. 2b). The plot indicates that ‘R’ and ‘bp’ are inversely related, namely noisier strains are also less reliable and exhibit higher phenotypic diversity (*right* y-axis). The plot reveals two additional characteristics. The first is that phenotypic diversity is persistently higher (>0.5) than bioprocessing noise (<0.5) for all cases. We attribute this to the different extracellular microenvironment between batch growth and microfluidics. During fermentation, the extracellular time invariance in microfluidics is replaced by limited mixing that induces microheterogeneities such as chemical gradients of key nutrients or waste products. These amplify intracellular bioprocessing noise during fermentation, enhancing population diversity in culture. Specifically for *Yarrowia lipolytica* at early exponential phase (Fig. 2b), the upper reliability (or diversity) limit set by the fermentation microenvironment was R_o_ ~ 0.5 at zero-amplitude intracellular fluctuations (bp = 0). The second feature is the non-linear dependence of reliability on bioprocessing noise as indicated by the exponential decay curve fitting in Fig. 2b. This nonlinear amplification of intracellular noise is likely due to low autoregulation between extracellular events with intracellular reactions, in contrast to the established feedback stabilizing mechanisms in gene networks^32^.

In conclusion, we employed microfluidics for comparing bioprocessing dynamics at the single-cell level in a controlled microenvironment with cell-to-cell bioprocessing diversity in culture during batch growth. Within a time-invariant microfluidic environment, we observed that lipogenesis is sporadic with time, rather than monotonic. Such an observation can only be achieved through the longitudinal analysis of single cells, otherwise impossible at the culture level, or through alternative single cell methods such as flow cytometry. In light of this, we introduced bioprocessing noise and identified its epigenetic origins. Subsequently, we compared the time-dependent single cell behavior in microfluidics with the persistent cell-to-cell variability during batch growth. The use of microfluidics was also critical here, by enabling deterministic control of the extracellular environment. Through this, we discovered that intracellular lipid production fluctuations are non-linearly linked with extracellular events, a critical insight to biofuels production, as well as medical implications of fatty acids, such as in liver disease^14^, adipogenesis^33^ and cancer^34^. Microfluidics has been crucial in screening gene and protein interactions and resulting complex phenotypes at the single cell level^35^,^36^. Our results expand the possibilities of such microsystems to identifying the true phenotype of a strain, which is otherwise masked by microheterogeneities within the extracellular environment. Additionally, such microsystems enable the accurate control or assessment of the extracellular microenvironment on the efficiency of metabolic processes, thus enabling enhanced bioprocess investigations and potentially optimized bioreactor designs.

## Materials and Methods

The experiments were performed using the two derivatives *Po1g* and *MTYL053* of the oleaginous yeast *Yarrowia lipolytica*. More detailed information on the auxotrophic wild type strain (*Po1g* – Leu^-^) and the engineered one (*MTYL053*) are included in reference^5^. The cells were grown at room temperature (regulated at 25 °C ± 1 °C) until early stationary phase in YPD media, with glucose as the carbon source (10 g/L Yeast Extract – Difco Laboratories, 20 g/L Bacto-Peptone – Difco and 20 g/L glucose – Sigma Aldrich). To visualize the lipid droplets, the bodipy dye (BODIPY® 493/503 (4,4-Difluoro-1,3,5,7,8-Pentamethyl-4-Bora-3a,4a-Diaza-*s*-Indacene – Molecular Probes) solution in DMSO (Molecular Probes) was added in YPD media at a concentration of 250 ng/ml, followed by a 4 hour long incubation under the same temperature and shaking conditions as those maintained during batch growth. Specific to the microfluidic experiments, following the 4 hour long staining period, the cells were transferred to a gastight syringe at a 10x dilution in YPD media containing a lower concentration of the bodipy dye (100 ng/ml) and 0.02% DMSO and loaded to the microfluidics through one inlet (Fig. S5a). Following immobilization, the same solution (YPD-Bodipy concentration 100 ng/ml, 0.02% DMSO) was continuously supplied through a second inlet at a rate of 1 μL/min to enable stable immobilization. On average ~n = 90 individual cells per strain and growth condition were studied.

The microfluidics were fabricated with conventional soft lithography by molding polydimethilsiloxane (PDMS, Sylgrad 184, Dow Corning) and bonding on a glass coverslip^37^. Briefly, a 10:1 monomer to catalyst ratio was manually mixed and degassed for 2 hour in a vacuum desiccator. The mixture was subsequently poured over a mask (SU8 photoresist on a silicon substrate) and baked for 2 hours at 70 °C, followed by cutting using a razor blade, plasma assisted bonding to glass coverslips and overnight baking at 70 °C. Two inlets were integrated, one for cell loading and one for media delivery; one outlet enabled waste collection (Fig. S5a). Media and cell flow were enabled by two syringe pumps (Harvard Apparatus) and Tygon tubing (inner diameter 0.02″).

The cell trapping design was based on the Tan and Takeuchi axial percolation filtration method and the principle of path of minimal hydrodynamic resistance^38^; briefly, 16 trapping sites were arrayed connected to a cell-loading and a media-delivering channel (50 μm wide – 10 μm thick). The traps comprised of a dwell chamber and a narrow indentation with approximate widths 6 μm and 2.5 μm respectively (Fig. S5b)^39^. Given the channel’s height being almost double than the cell’s, we anticipate that fluid flow also occurs through the narrow indentation even when it is infilled with an individual cell. On average, 6–8 cells were trapped per experiment, resulting in an approximately 50% trapping efficiency; however, such a reduced trapping efficiency did not limit our experimental throughput, due to temporal limitations placed by optical sampling. The latter necessitated approximately 1–1.5 min dwell time to complete the confocal imaging at three wavelength channels, namely green excitation for lipid detection, red excitation for viability detection and brightfield for cell size determination. Such dwell times imposed the upper limit to the temporal resolution of the lipid expression fluctuation analysis, which we accommodated by selecting a time step of 20 min for the whole cell array (more information is included in the Supplementary Information). The cells at late stationary phase remained viable for more than 9 hours. This was determined by adding the propidium iodide dye (Sigma Aldrich) in the media (Fig. S3). Confocal fluorescent imaging was employed to determine the lipid content in individual cells. This imaging modality, contrary to higher resolution and specificity approaches, such as Electron Microscopy, enabled long term longitudinal imaging of individual cells under minimal perturbations to cell physiology. Further information on cell growth, staining, imaging and data analysis, as well as supplementary images are available within the online Supplementary Information.

## Additional Information

**How to cite this article**: Vasdekis, A. E. *et al.* Origins of Cell-to-Cell Bioprocessing Diversity and Implications of the Extracellular Environment Revealed at the Single-Cell Level. *Sci. Rep.* **5**, 17689; doi: 10.1038/srep17689 (2015).

## Supplementary Material

Supplementary Information
