# Supplementary material for: Origins of Cell-to-Cell Bioprocessing Diversity and Implications of the Extracellular Environment Revealed at the Single-Cell Level
Source: Sci Rep. 2015 Dec 14;5:17689. doi: 10.1038/srep17689 (PMC4677318; doi:10.1038/srep17689)
Supplement: Supplementary Information [file srep17689-s1.pdf]

Supplementary Information

Origins of Cell-to-Cell Bioprocessing Diversity and Implications of the Extracellular Environment Revealed at the Single-Cell Level

A. E. Vasdekis[1,2,*], A. M. Silverman[3], G. Stephanopoulos[3]

[1] Department of Physics, University of Idaho, Moscow, ID, 83844, USA.

[2] Molecular Sciences Laboratory, Pacific Northwest National Laboratory, Richland, WA, 99354, USA.

[3] Department of Chemical Engineering, Massachusetts Institute of Technology, Cambridge, MA 02139, USA.

Materials and Methods

Strains and Media: All experiments were performed using the two derivatives *Po1g* and *MTYL053* of the oleaginous yeast *Yarrowia Lipolytica*. Where indicated, cycloheximide (CHX - Sigma Aldrich) was added to the media during batch growth for image cytometry, or in the media flowing in the microfluidics following immobilization (see below) at a concentration of 33 µg/ml (similar to [3, 4]). CHX was dissolved in DMSO (Molecular Probes) at a 330 mg/ml concentration, the highest possible concentration for completely dissolving the compound. The CHX solution was divided in 20 µL aliquots and stored at -20°C until further use. DMSO was chosen for dissolving CHX as the lipid stain was dissolved in the same solvent (see below). It is worth noting that DMSO has been shown to affect yeast physiology by inducing growth inhibition and cell-wall permeability [5, 6]; however, in these reports no changes were detected for DMSO concentrations equal to or less than 1% in YPD media. In our experiments, we observed no growth variations for *Yarrowia Lipolytica* at 0.2% DMSO concentration in YPD (Fig. S2). Despite its significant advantage in improving the optical signal to noise ratio, we employed an even lower DMSO concentration than the abovementioned physiological limit by an order of magnitude (0.02%). In comparing *Po1g* with or without CHX and *MTYL053*, care was taken so that the total DMSO concentration in the media was the same in all cases (0.02%).

Cell Growth and Sampling: Cells were grown at room temperature (regulated at 25C±1C) under orbital shaking conditions (180 rpm). For this, cells were transferred to sterile Falcon tubes containing 5 ml of YPD media at a 50x dilution. In all experiments, the cells were harvested during early stationary phase at 24 hours (OD_{600} ~ 1.5 – Fig. S2) prior to inoculation. At this time-point, the cells exhibit an enhanced lipid content, as well as a reduced probability for division in comparison to earlier growth stages. The former was an advantage for imaging intracellular neutral

lipids, while the latter was particularly pertinent in single-cell immobilization in microfluidics, addressing the potential of device clogging during exponential growth. Following microfluidic immobilization, cells were occasionally observed to undergo budding during time-lapse confocal imaging with a probability of approximately 10% (Fig. S4); due to the low lipid content and associated low optical signal to noise ratio, such cases were discarded from our study.

Sample Preparation: For both the single-cell analysis in microfluidics and image cytometry, a 1.5 ml cell suspension (1x dilution) was collected following 24 hours of continuous growth. As discussed within the main text, to fluorescently visualize the lipid droplets, a bodipy dye (BODIPY® 493/503 (4,4-Difluoro-1,3,5,7,8-Pentamethyl-4-Bora-3a,4a-Diaza-*s*-Indacene – Molecular Probes) solution in DMSO (Molecular Probes) was added in YPD media at a concentration of 250 ng/ml, followed by a 4 hour long incubation. For image cytometry, we sampled 50 μL from the cell suspension, deposited them on a coverslip, and covered it on top by a second coverslip to minimize cell motion and out-of-focus contributions. Specific to the microfluidic experiments, following the 4 hour long staining period, the cells were transferred to a gastight syringe at a 10x dilution in YPD media containing a lower concentration of the bodipy dye (100 ng/ml) and 0.02% DMSO and loaded to the microfluidics through one inlet (Fig. S5a). Following immobilization, the same solution (YPD-Bodipy concentration 100 ng/ml, 0.02% DMSO) was continuously supplied through a second inlet at a rate of 1 μL/min to enable stable immobilization. A 40 ng/ml propidium iodide solution in water (Sigma Aldrich) was included in the media during immobilization to detect cell apoptosis and death. No such phenomena were observed however during the fluctuation analysis experiments. The cells at late stationary phase remained viable for more than 9 hours. This was determined through the propidium iodide dye (Sigma Aldrich) in the media (Fig. S3).

Imaging: Vesicle photonics through confocal fluorescent microscopy was employed for visualizing the stained lipid droplets [10]. To this end, an inverted microscope (Leica DMI6000) coupled to a spinning disk confocal system (Yokogawa, CSU10) was employed. A two wavelength laser illumination (488 nm for lipids and 532 nm for viability and apoptosis detection) as well as bright-field imaging (for cell size) were employed. Images were acquired through a 100x oil objective (100x/1.4 NA) on a CCD array (Photometrics CoolSNAP HQ2, pixel size 6.45 μm x 6.45 μm) with typical acquisition settings of a 300 msec integration time, ~10 mW optical excitation on the sample for both lines and a z-scanning step of ~250 nm. Confocal imaging was essential for enhanced imaging contrast and distinguishing lipid droplets in 3D since occasionally LDs overlapped along the optical sectioning path (z-axis) and were thus indistinguishable with conventional epifluorescence microscopy (Fig. S6). Neutral lipid expression was sampled on average 10 times every 20 min. The lipid content of individual cells (S_i) was determined through the ratio of the product between the number and area of cytosolic lipid droplets over the cell size as determined by bright field microscopy.

Each cell required approximately 1-1.5 min of optical sampling for all three wavelength channels. This imposed an upper limit to the temporal resolution of the lipid expression fluctuation analysis, which we accommodated by selecting a time step of 20 min for the whole cell array. Under such imaging and staining conditions, the smallest detectable lipid droplet was of an area equal to 0.09 μm^2. This limit was set by requiring a signal to noise ratio > 2 with the background primarily stemming from intracellular fluorescence (Fig. S7). In the context of spatial resolution (~200 nm according to the manufacturer), a single dark pixel criterion was used to distinguish two lipid droplets.

Due to the lower axial resolution (z-axis) of the microscope than the lateral one (xy-plane), we quantified the size of the lipid droplets using the maximum intensity projection analysis rather than direct 3D confocal imaging [11]. This technique also enabled sparser optical sampling along the z-axis, thus minimizing bleaching and phototoxicity. A comparison between the maximum intensity projection analysis and direct 3D confocal imaging of lipid droplets yielded that the estimated lipid content per cell scaled linearly between the two methods with an approximate 9% overestimation in the latter case (Fig. S8).

Following acquisition, the images were stored for processing and analysis using ImageJ (National Institutes of Health). For processing, the 3D confocal images were converted to 2D through the maximum intensity projection analysis, followed by convolution with a Gaussian (radius of decay = standard deviation – σ) to attenuate its low spatial-frequency components. Subsequently a bandpass filter was applied to the images (filter size equal to maximum feature size), followed by subtracting the filtered image from the original one (droplet-finder plug-in, ImageJ). The lipid droplets were then identified by applying thresholding based on the histogram entropy criterion [12]. In this way, the number and area of individual lipid droplets were determined and normalized over the cell area as determined by bright field imaging.

Data Analysis: Due to the presence of outliers in our observations (some cells exhibiting an increased lipid content), robust statistics were employed in data analysis (see for example [13]). To this end, a custom script was written in Matlab for performing the statistical analysis for the longitudinal fluctuations and noise determination. Linear fits and correlation analyses were performed in Origin Pro (OriginLab, 64Bit, 2015).

Supplementary Figures

Fig. S1

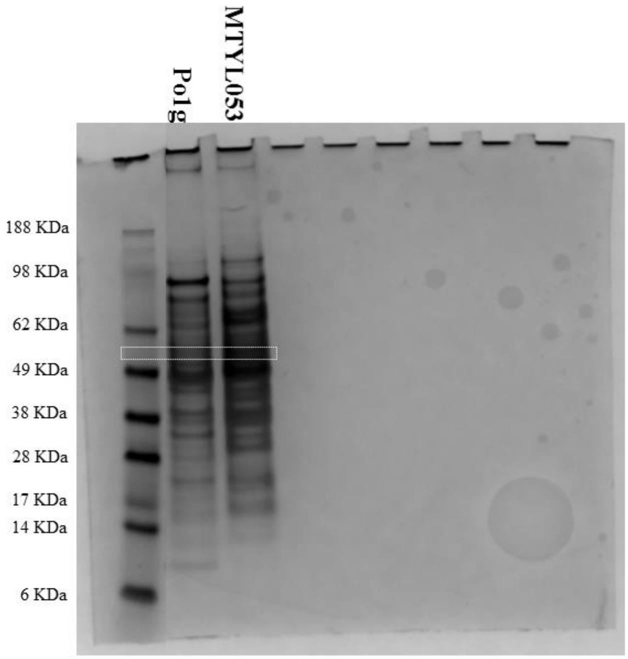

SDS-PAGE gel demonstrating in a qualitative fashion the protein overexpression in *MTYL053* compared to *Po1g*, specifically around 50 kDa, approximately the molecular mass of DGA1 [14]. For obtaining the gel, the cells were grown for 24 hours in YPD media, including 0.02% DMSO (the same concentration as in the microfluidic and image cytometry single cell experiments). Both *MTYL053* and *Po1g* reached the same optical density during this time. For lysis, the cells were centrifuged at 4°C, 10,000 x g for 15 minutes and pelleted. All supernatant was removed but ~100 µL, 200 µL of buffer (100 mM NH_4HCO_3) were added, followed by a vortex step. The resuspended pellet was transferred to a biopulverizer (BioSpec Products) with Liquid N_2, sample "beads" were introduced and pulverized with hammer and transferred to 2 mL Eppendorf BioPur microcentrifuge tube. A BCA protein assay was performed to obtain the samples' volume and masses. The sample was incubated at 60°C for 30 min using a thermomixer at 800 rpm and washed into 50 mM NH_4HCO_3 for SDS-PAGE (experiments performed by Heather Brewer and Larissa Iracheta – Pacific Northwest National Laboratory).

Fig. S2

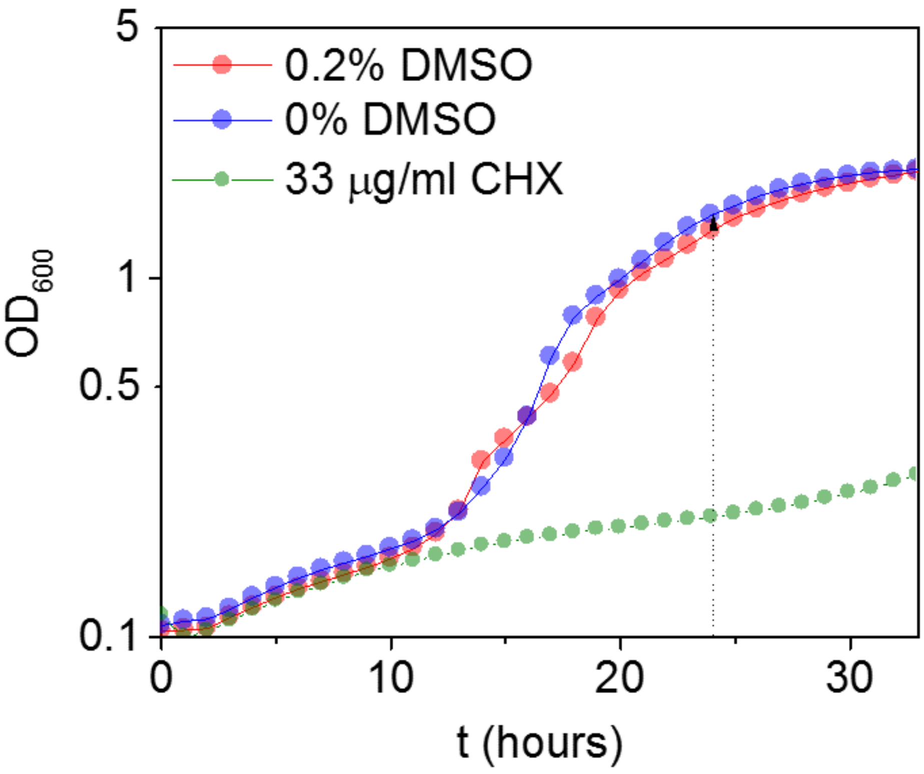

Growth curves measured for *Po1g* in YPD media at two DMSO concentrations and cycloheximide (CHX). The growth curves were determined through the optical turbidity at a wavelength of 600 nm at 0% DMSO (blue line, open dots), 0.2% DMSO (red line, dots) and 33 μg/ml CHX and similar DMSO concentration (green line, dots). The vertical dotted line denotes the cell harvesting time point within the early stationary phase, at 24 hours. The measurements were performed in a 96-well plate using a Bioscreen C Pro instruement; each measurement represents an average of four independent wells.

Fig. S3

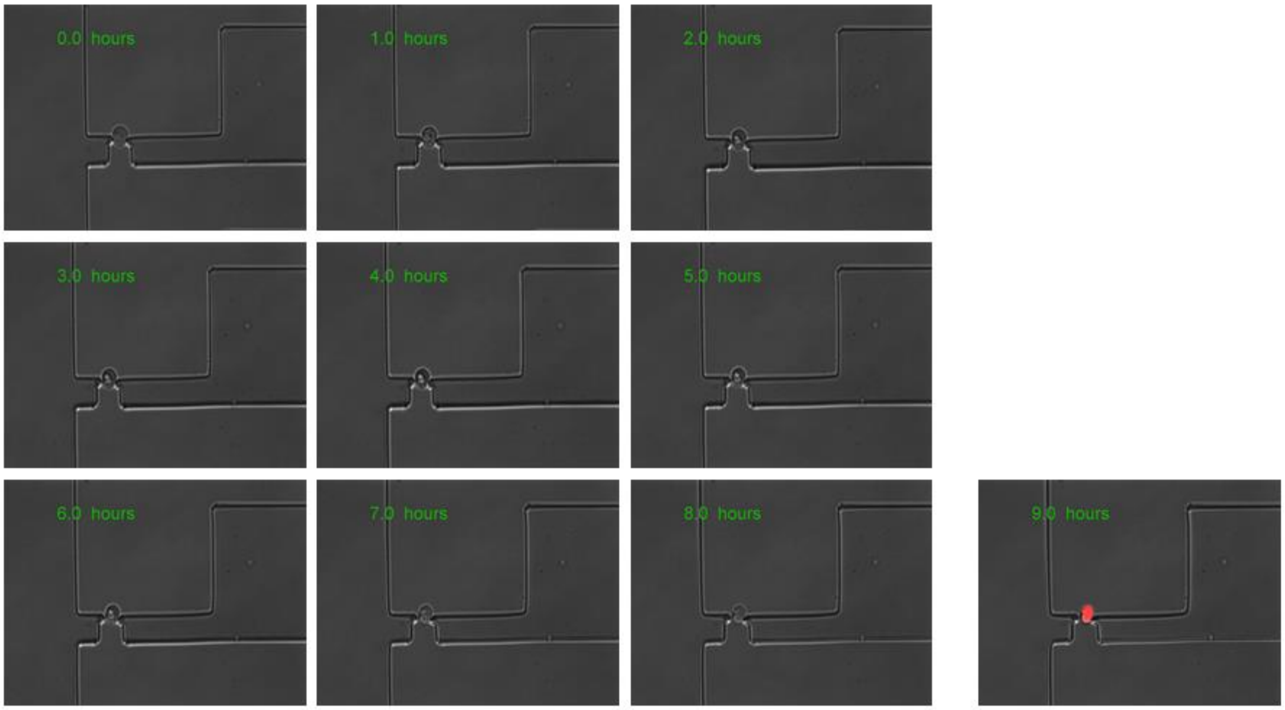

A single *Yarrowia Lipolytica po1g* cell immobilized in a microfluidic trap; propidium iodide was added in the continously supplied media (see Materials and Methods) to evaluate the state and viability of the cell. Cells remained viable for many hours (up to 9 hours shown in the figure) before becoming apoptotic and thus enabling the internalization of the dye. Propidium iodide was employed in our single cell fluctuation experiments and co-registered in a different fluorescent channel than the bodipy lipid stain (see Materials and Methods).

Fig. S4

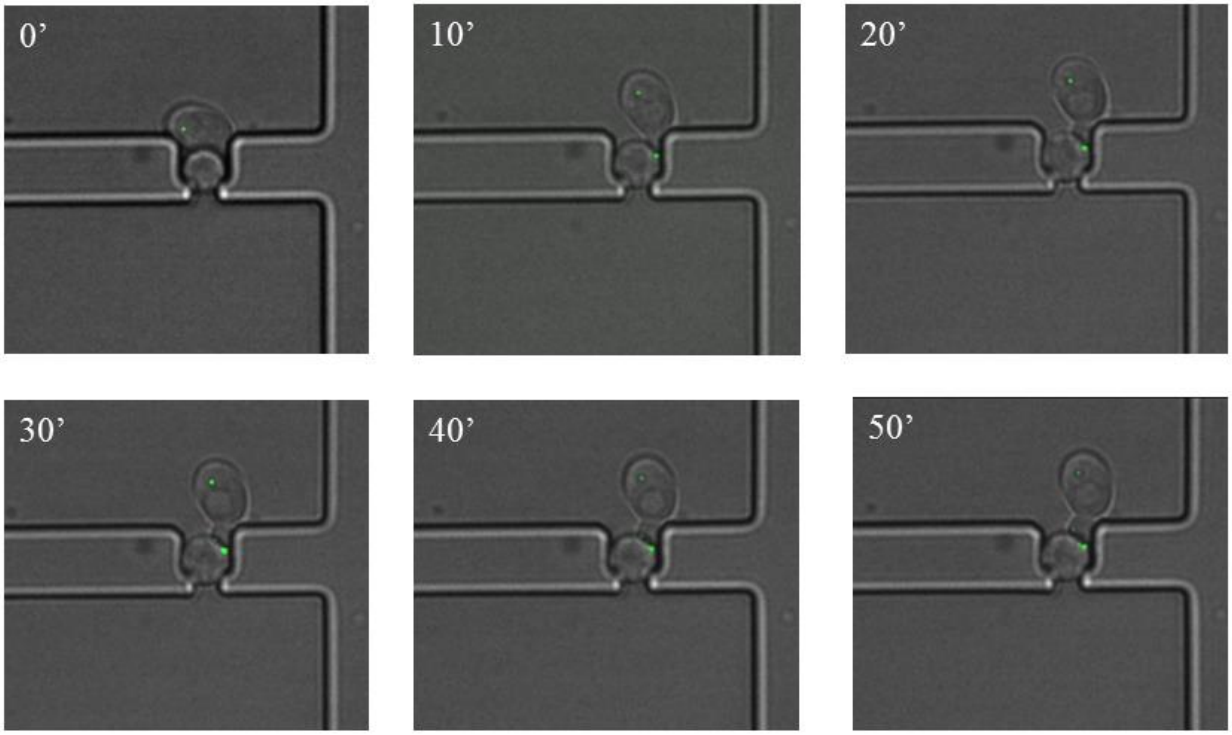

Yarrowia Lipolytica Po1g undergoing division under microfluidic immobilizaiton conditions and confocal imaging of lipid droplets. YPD media are continuously supplied at a rate of 1 µL/min, including the bodipy dye at a concentration of 800 ng/ml (4x higher than typically used in the fluctuation analysis) in 0.2% DMSO (similarly 10x higher than typically used in the fluctuation analysis). The cells were collected at an early stationary phase (24 hours), thereby enforcing a low probability of undergoing budding (~10%) due to ageing. Budding cells were discarted due to their reduced lipid content.

Fig. S5

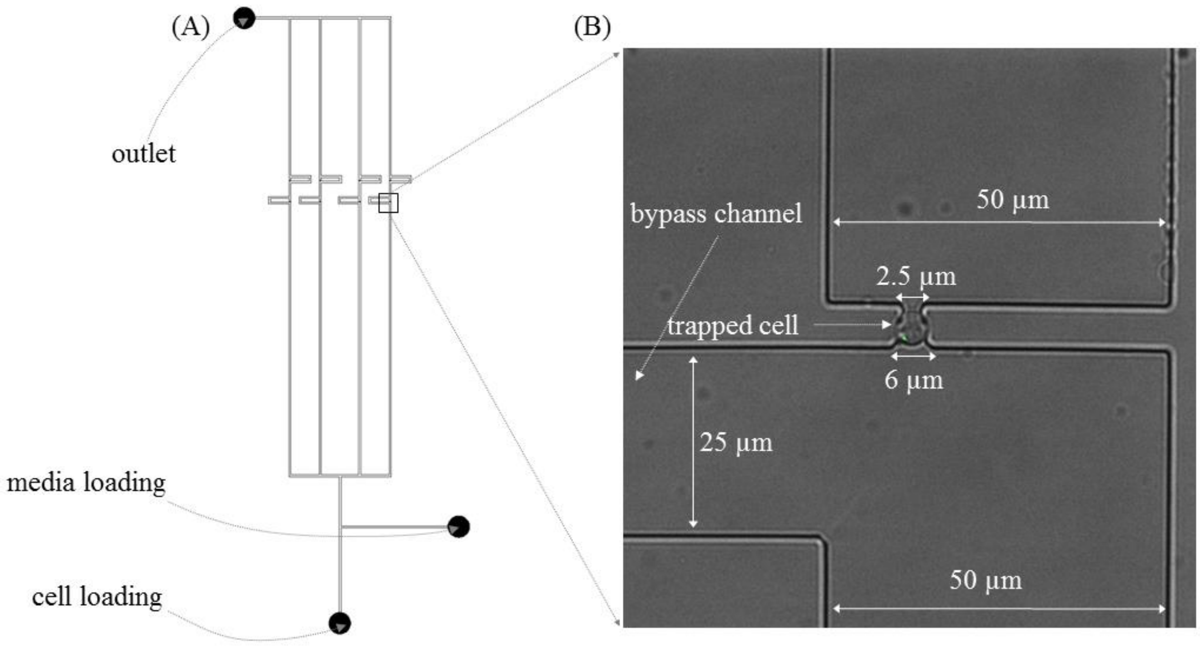

(a) A schematic illustrating the cell-trapping microfluidic system, along with the two inlets for cell and media loading and one outlet for waste collection (b) Microfluidic trap for *Yarrowia Lipolytica Po1g*, microfabricated in PDMS. Cells flow from the bottom of the image in 50 µm wide channels (~ 10 µm tall). Cells get trapped at the 6 µm wide dwell chamer, unable to flow through the 2.5 µm wide indentation. Subsequent cells follow the flow-through path to the left, through a 25 µm wide channel. Once the trapping is terminated, media flow through the same delivery channel at a rate of 1 µL/min.

Fig. S6

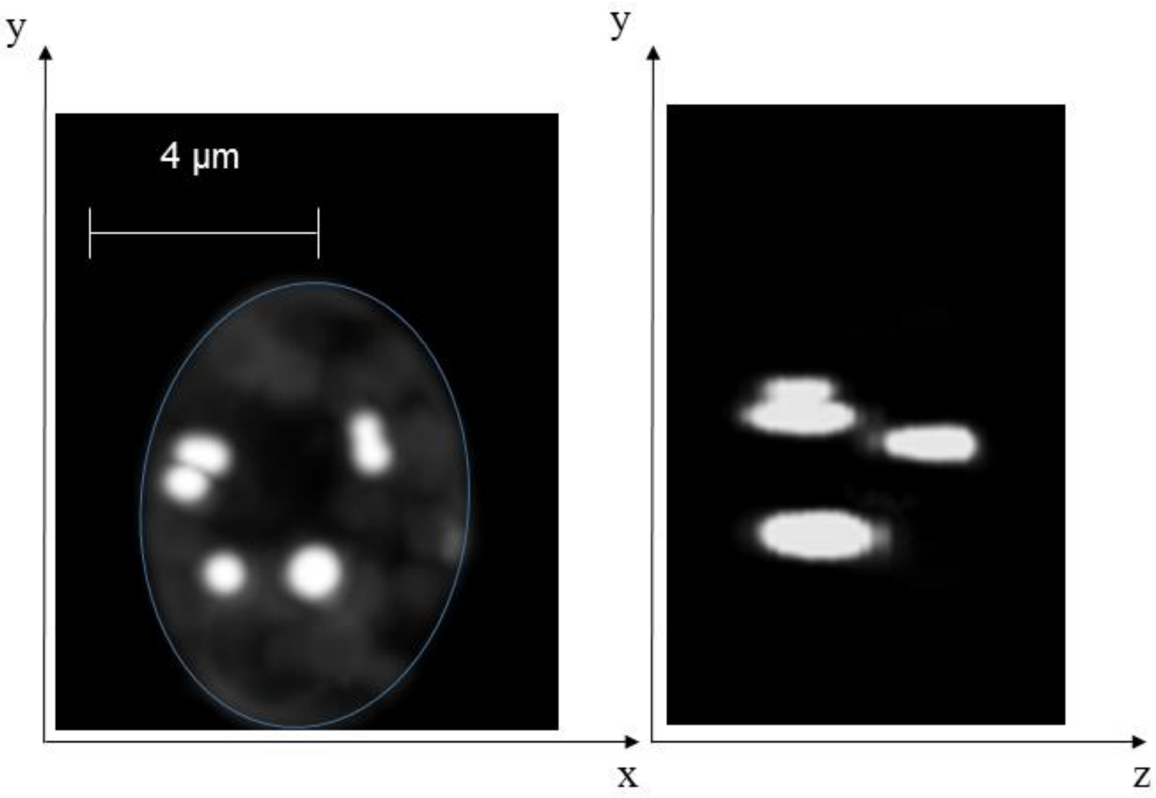

A single *Po1g* cell imaged with spinning disk confocal microscopy; the cell wall is highlighted through a dotted blue line in the xy plane (maximum intensity projection analysis - *left*). On the *right* a section along the optical path (z-axis) is shown, highlight the vertical overlap of one lipid droplet with a manifold of two.

Fig. S7

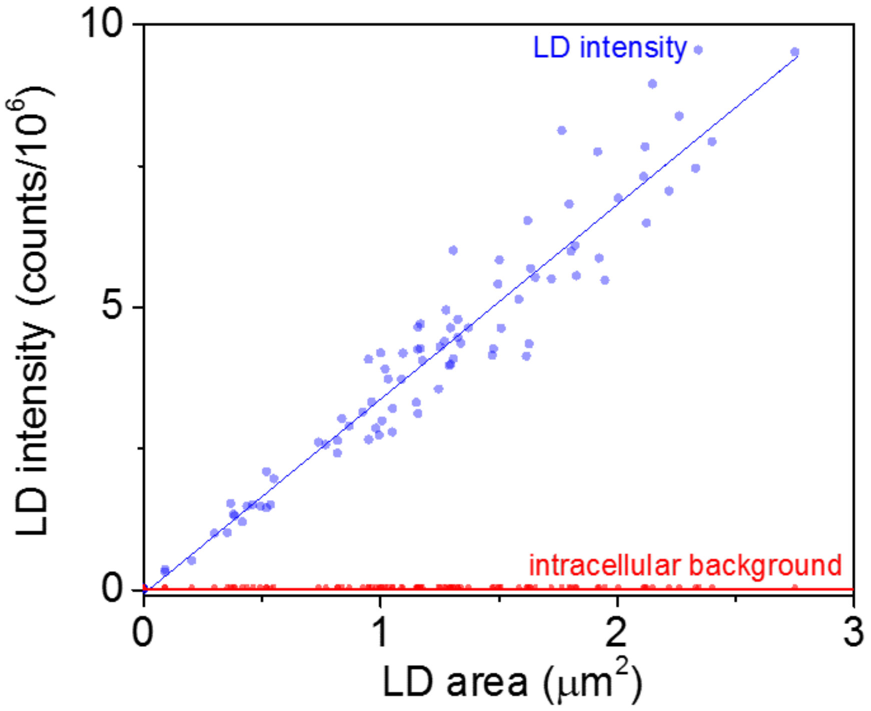

The relationship between the intensity (y-axis) and area (x-axis) of individual lipid droplets (blue dots), including a linear fit with a r = 0.97 Pearson's coefficient (blue line). In the same experiment, the intracellular background fluorescence was determined and shown in red dots. The imaging and staining conditions are discussed in the Supplementary Materials (bodipy dye concentration 100 ng/ml).

Fig. S8

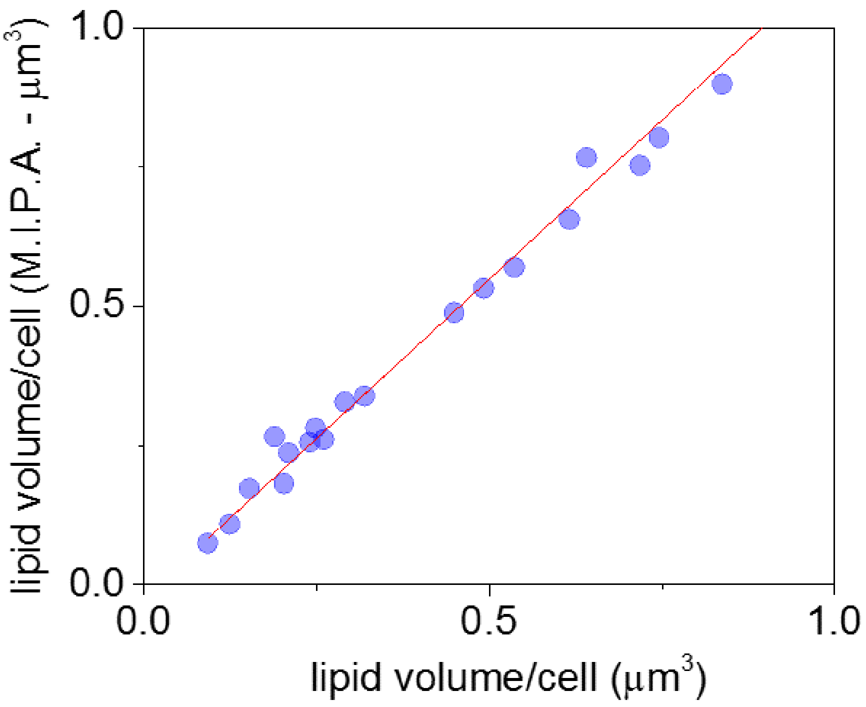

The experimentally determined linearity between the single-cell lipid content determined by direct 3D confocal imaging (x-axis) and indirect 2D images through the maximum intensity projection analysis (M.I.P.A.). For the latter, the following experssion was employed to convert 2D areas (circles) to 3D volumes (spheres): volume $= 2.4 * [\text{area}]^{3/2}$. The linear fit exhibits a Pearson's coefficient of r = 0.99 and a slope of 1.09, indicating a constant 9 % overestimation of lipid content using the maximum intensity projection analysis.

Supplementary References

1. Frenzen, C.L. and P.K. Maini, *Enzyme Kinetics for a 2-step Enzymic Reaction with Comparable Initial Enzyme-Substrate Ratios.* Journal of Mathematical Biology, 1988. **26**(6): p. 689-703.
2. Tai, M. and G. Stephanopoulos, *Engineering the push and pull of lipid biosynthesis in oleaginous yeast Yarrowia lipolytica for biofuel production.* Metabolic Engineering, 2013. **15**: p. 1-9.
3. Belle, A., et al., *Quantification of protein half-lives in the budding yeast proteome.* Proceedings of the National Academy of Sciences of the United States of America, 2006. **103**(35): p. 13004-13009.
4. Schneider-Poetsch, T., et al., *Inhibition of eukaryotic translation elongation by cycloheximide and lactimidomycin.* Nature Chemical Biology, 2010. **6**(3): p. 209-217.
5. Murata, Y., et al., *Dimethyl sulfoxide exposure facilitates phospholipid biosynthesis and cellular membrane proliferation in yeast cells.* Journal of Biological Chemistry, 2003. **278**(35): p. 33185-33193.
6. Sadowska-Bartosz, I., et al., *Dimethyl sulfoxide induces oxidative stress in the yeast Saccharomyces cerevisiae.* Fems Yeast Research, 2013. **13**(8): p. 820-830.
7. Thorsen, T., S.J. Maerkl, and S.R. Quake, *Microfluidic large-scale integration.* Science, 2002. **298**(5593): p. 580-584.
8. Tan, W.-H. and S. Takeuchi, *A trap-and-release integrated microfluidic system for dynamic microarray applications.* Proceedings of the National Academy of Sciences of the United States of America, 2007. **104**(4): p. 1146-1151.
9. Vasdekis, A.E., *Single microbe trap and release in sub-microfluidics.* Rsc Advances, 2013. **3**(18): p. 6343-6346.
10. Vasdekis, A.E., et al., *Vesicle Photonics.* Annual Review of Materials Research, Vol 43, 2013. **43**: p. 283-305.
11. Chumnanpuen, P., et al., *Lipid biosynthesis monitored at the single-cell level in Saccharomyces cerevisiae.* Biotechnology Journal, 2012. **7**(5): p. 594-601.
12. Sahoo, P.K., et al., *A survey of thresholding techniques.* Computer Vision Graphics and Image Processing, 1988. **41**(2): p. 233-260.
13. Committee, A.M., *Robust statistics: a method of coping with outliers.* AMC Technical Briefs, 2001. **6**.
14. Stoveken, T., et al., *The wax ester synthase/acyl coenzyme A : diacylglycerol acyltransferase from Acinetobacter sp strain ADP1: Characterization of a novel type of acyltransferase.* Journal of Bacteriology, 2005. **187**(4): p. 1369-1376.
